# Development and Validation of a Nomogram to Predict Distant Metastasis in Elderly Patients With Renal Cell Carcinoma

**DOI:** 10.3389/fpubh.2021.831940

**Published:** 2022-01-28

**Authors:** Jinkui Wang, Chenghao Zhanghuang, Xiaojun Tan, Tao Mi, Jiayan Liu, Liming Jin, Mujie Li, Zhaoxia Zhang, Dawei He

**Affiliations:** ^1^Department of Urology, Chongqing Key Laboratory of Children Urogenital Development and Tissue Engineering, Chongqing Key Laboratory of Pediatrics, Ministry of Education Key Laboratory of Child Development and Disorders, China International Science and Technology Cooperation Base of Child development and Critical Disorders, National Clinical Research Center for Child Health and Disorders, Children's Hospital of Chongqing Medical University, Chongqing, China; ^2^Department of Urology, Yunnan Key Laboratory of Children's Major Disease Research, Kunming Children's Hospital (Children's Hospital Affiliated to Kunming Medical University), Kunming, China; ^3^Department of Urology, Nanchong Central Hospital, The Second Clinical Medical College, North Sichuan Medical University, Nanchong, China

**Keywords:** nomogram, renal cell carcinoma, risk, distant metastasis, SEER

## Abstract

**Background:**

Renal cell carcinoma (RCC) is the most common renal malignant tumor in elderly patients. The prognosis of renal cell carcinoma with distant metastasis is poor. We aim to construct a nomogram to predict the risk of distant metastasis in elderly patients with RCC to help doctors and patients with early intervention and improve the survival rate.

**Methods:**

The clinicopathological information of patients was downloaded from SEER to identify all elderly patients with RCC over 65 years old from 2010 to 2018. Univariate and multivariate logistic regression analyzed the training cohort's independent risk factors for distant metastasis. A nomogram was established to predict the distant metastasis of elderly patients with RCC based on these risk factors. We used the consistency index (C-index), calibration curve, and area under the receiver operating curve (AUC) to evaluate the accuracy and discrimination of the prediction model. Decision curve analysis (DCA) was used to assess the clinical application value of the model.

**Results:**

A total of 36,365 elderly patients with RCC were included in the study. They were randomly divided into the training cohort (*N* = 25,321) and the validation cohort (*N* = 11,044). In the training cohort, univariate and multivariate logistic regression analysis suggested that race, tumor histological type, histological grade, T stage, N stage, tumor size, surgery, radiotherapy, and chemotherapy were independent risk factors for distant metastasis elderly patients with RCC. A nomogram was constructed to predict the risk of distant metastasis in elderly patients with RCC. The training and validation cohort's C-indexes are 0.949 and 0.954, respectively, indicating that the nomogram has excellent accuracy. AUC of the training and validation cohorts indicated excellent predictive ability. DCA suggested that the nomogram had a better clinical application value than the traditional TN staging.

**Conclusion:**

This study constructed a new nomogram to predict the risk of distant metastasis in elderly patients with RCC. The nomogram has excellent accuracy and reliability, which can help doctors and patients actively monitor and follow up patients to prevent distant metastasis of tumors.

## Background

Renal cell carcinoma (RCC) is the most common renal malignant tumor in adults, accounting for about 3% of all human tumors (1), with more than 400,000 newly diagnosed patients each year (2). The proportion of elderly patients over 65 years old in RCC is more than 70% (3). Moreover, due to the increase of population aging and the extension of life expectancy caused by improved medical levels, the proportion is still rising (1).

According to the tumor metastasis, RCC is divided into metastatic RCC (mRCC) and non-metastatic RCC (nmRCC). Tumor metastasis is the critical factor to determine the prognosis of RCC patients. The prognosis of patients with mRCC is poor, and the median survival time is only 10.2 months (4), while the prognosis of nmRCC is good, and it is even considered to be cured entirely (5). Studies have shown that 18–30% of RCC patients have distant metastasis at the first diagnosis (6). In comparison, 33% of patients have recurrence and metastasis after surgical excision of the tumor, and it is unclear which patients are prone to metastasis (7).

The main sites of RCC metastasis were lung and bone, accounting for 43.6 and 27.6% of mRCC, respectively. Brain and liver metastasis accounted for 4.4%, respectively (8). Data processing technology has been continuously improved by continuously updating databases such as surveillance, epidemiology, and final results (SEER) of the National Cancer Institute of the United States. The nomograms prediction model represented by UISS (9), SSIGN (10), Leibovich (11) was born and provided treatment suggestions for clinicians. In terms of the RCC transfer prediction model, we found that the prediction models for each leading transfer site of RCC were reported (12–14), but the C-index of the model was 0.714–0.803, and the area under the curve (AUC) was 0.767–0.780, indicating that the relative accuracy was insufficient. And the distant metastasis prediction model for the overall large sample of the elderly has not been reported.

At present, artificial intelligence has been widely used in human health. Dhanamjayulu et al. (15) used real-time image processing and machine learning to identify malnutrition, predict BMI from facial images, and identify unhealthy people quickly. Gadekallu et al. (16, 17) used the deep learning model for the early detection of diabetic retinopathy. And the nomogram is the most commonly used prediction model. To improve the prognosis of elderly patients with RCC, we developed a risk prediction model for distant metastasis of elderly RCC. We validated its accuracy to provide treatment guidance for clinical work.

## Method

### Data Source and Data Extraction

The patient information was extracted from the Surveillance, Epidemiology, and End Results (SEER) project of the National Cancer Institute in the United States to include all elderly patients with RCC from 2010 to 2018. SEER database is the National Cancer Database of the United States, including 18 cancer registration centers, covering about 28% of the national population (18). Demographic information, clinicopathological information, and follow-up data of cancer patients can be downloaded from the SEER database. Access to http://seer.cancer.gov/ provides all the data for this study. Since the patient information in the SEER database is public and anonymous, our research does not require ethical approval and patients' informed consent. Our research method conforms to the research criteria of SEER data release.

**Figure 1:**
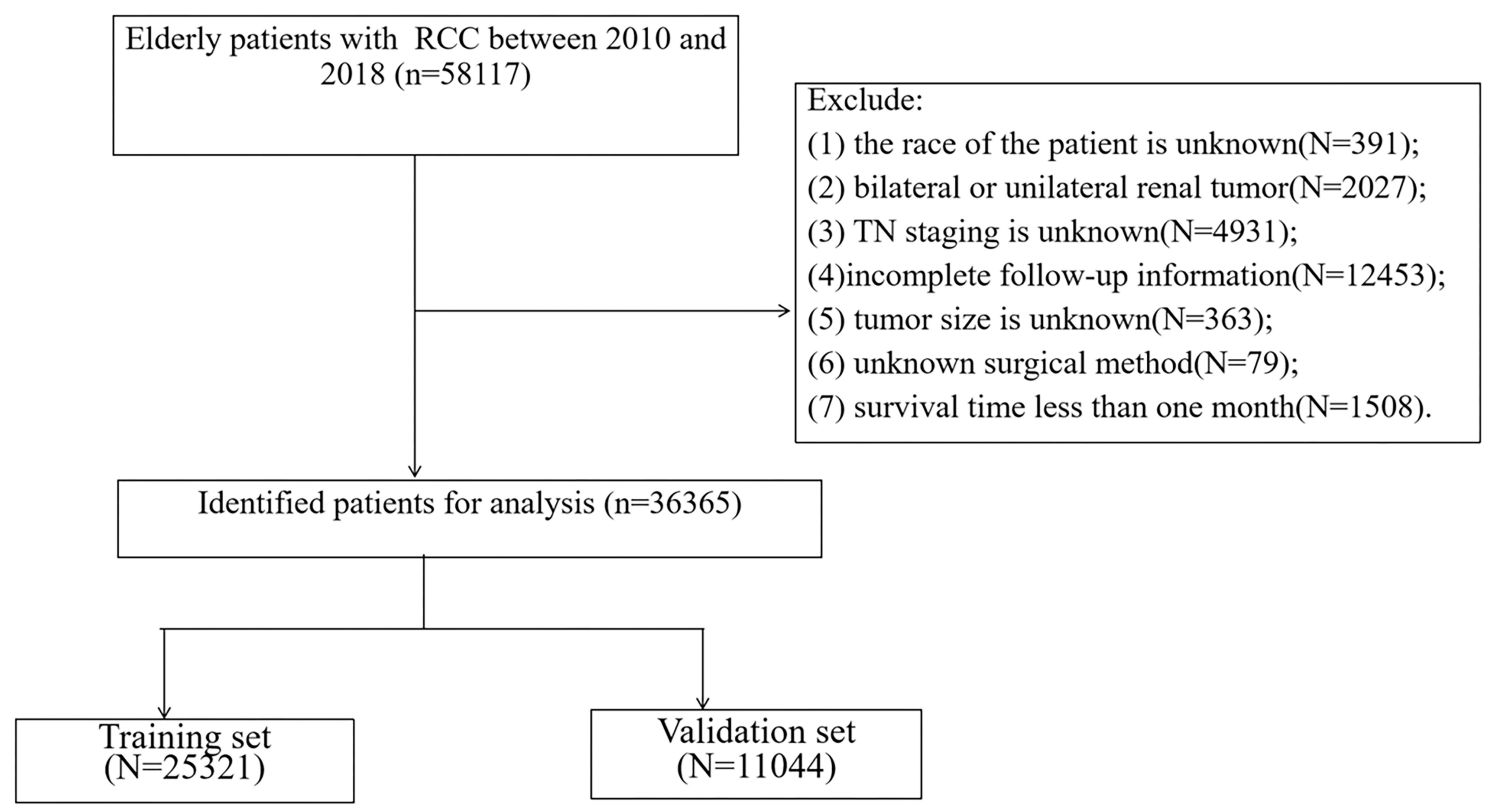
The flowchart of including and dividing patients.

We collected patient information, including age, sex, race, year of diagnosis, marital status, tumor histological type, histological grade, tumor side, tumor size, T stage, N stage, surgical method, radiotherapy, and chemotherapy. Inclusion criteria: (1) pathological diagnosis of RCC (ICD-O-3 code 8260, 8310, 8312, 8317); (2) Aged 65 or above; (3) The years of diagnosis were 2010–2018. Exclusion criteria: (1) the race of the patient is unknown; (2) bilateral or unilateral renal tumor; (3) TN staging is unknown; (4) incomplete follow-up information; (5) tumor size is unknown; (6) unknown surgical method; (7) survival time <1 month. The patient screening flow chart is shown in Figure 1.

Patients' race was divided into three categories: white, black, and others (American Indian/AK Native, Asian/Pacific Islander). The patients' years of diagnosis were divided into 2010–2014, 2015–2018. Marital status is divided into married and unmarried (including single, divorced, and widowed). Histological tumors include renal clear cell carcinoma, renal papillary cell carcinoma, chromophobe cell carcinoma, and some unclassified RCC. The histological grading of tumors is classified as grades I–IV, which are well-differentiated, moderately differentiated, poorly differentiated, and undifferentiated. Surgical procedures were classified as local excision of tumor (SEER codes 10–27), partial nephrectomy (SEER codes 30), and radical nephrectomy (SEER codes 40–80).

### Construction and Validation of the Nomogram

All patients enrolled were randomly assigned to either the training cohort (70%) or the validation cohort (30%). In the training cohort, univariate, and multivariate logistic regression models were used to analyze the independent risk factors for patient metastasis, and hazard ratio (HR) and 95% confidence interval (CI) were recorded simultaneously. The independent risk factors screened were used to establish a nomogram to predict the risk of distant metastasis in elderly patients with RCC. We validated the accuracy of the prediction model of the training cohort and the validation cohort by the calibration curve. We used the consistency index (C-index) to demonstrate the discrimination ability of the model. Meanwhile, We used the area under the receiver operating curve (AUC) to validate the model's accuracy.

### Clinical Utility

Decision curve analysis (DCA) was used to validate the clinical value of the model. DCA is a new algorithm to evaluate the net benefit value of the model under different thresholds (19). We also used DCA to compare the ability of the nomogram and TN staging to predict patients' risk of distant metastasis. In addition, we used a risk stratification system to divide all patients into high-risk and low-risk groups based on their nomogram scores. Log-rank test and Kaplan-Meier (K-M) curve were used to compare the survival and prognosis of patients in different risk groups.

### Statistical Analysis

Frequency description (%) was used for counting data, and the chi-square test or non-parametric *U*-test was used to compare groups. Measurement data (age, tumor size) were described using mean and standard deviation, and non-parametric *U*-tests were used to compare groups. Univariate and multivariate logistic regression models analyzed the risk factors of distant metastasis. The K-M curve and log-rank test compared the survival differences between groups. All statistical analyses were performed using R software 4.1.0 (http://www.Rproject.org) and SPSS 26.0 (IBM, Chicago, IL, USA). *P* < 0.05 was considered statistically significant.

## Results

### Clinical Features

we enrolled 36,365 elderly patients with RCC based on inclusion and exclusion criteria. The mean age of the patients was 73.4 ± 6.58 years, and 29,891 (82.2%) were white, 22,760 (62.6%) were male, and 21,817 (60.0%) were married. There were 2,772 patients (7.62%), 12,432 patients (34.2%), 7,630 patients (21.0%), and 1,778 patients (4.89%) of histological tumor grade I-IV, and 20,279 patients (55.8%) of tumor histological type RCC. Papillary cell carcinoma was 5,207 (14.3%), and chromophobe cell carcinoma was 1,855 (5.10%). The mean tumor size was 49.7 ± 34.5 mm. T stage included 17,089 (47.0%) T1a, 8,320 (22.9%) T1b, 3,475 (9.56%) T2, 7,287 (20.0%) T3, and 194 (0.53%) T4. Thirty-four thousand seven hundred and fifteen (95.5%) patients had stage N0. There were 7,254 (19.9%) patients without surgery, 3,083 (8.48%) patients with local tumor excision, 3,083 (8.48%) patients with partial nephrectomy, and 16,266 (44.7%) patients with radical nephrectomy. There were 2,269 (6.24%) patients who received chemotherapy and 1,105 (3.04%) patients who received radiotherapy. The clinicopathological information of all patients is shown in Table 1. There was no significant difference between the training cohort and the validation cohort.

**Table 1:** Clinicopathological information in elderly patients with renal cell carcinoma.

	**ALL**	**Training cohort**	**Validation cohort**	***p***
	***N* = 36,365**	***N* = 25,321**	***N* = 11,044**	
Age	73.4 (6.58)	73.4 (6.58)	73.3 (6.58)	0.227
Race				0.980
White	29,891 (82.2%)	20,807 (82.2%)	9,084 (82.3%)	
Black	4,279 (11.8%)	2,982 (11.8%)	1,297 (11.7%)	
Other	2,195 (6.04%)	1,532 (6.05%)	663 (6.00%)	
Sex				0.677
Male	22,760 (62.6%)	15,866 (62.7%)	6,894 (62.4%)	
Female	13,605 (37.4%)	9,455 (37.3%)	4,150 (37.6%)	
Year of diagnosis				0.054
2010–2014	18,953 (52.1%)	13,112 (51.8%)	5,841 (52.9%)	
2015–2018	17,412 (47.9%)	12,209 (48.2%)	5,203 (47.1%)	
Marital				0.355
No	14,548 (40.0%)	10,170 (40.2%)	4,378 (39.6%)	
Married	21,817 (60.0%)	15,151 (59.8%)	6,666 (60.4%)	
Histologic type				0.337
Clear cell	20,279 (55.8%)	14,113 (55.7%)	6,166 (55.8%)	
Papillary	5,207 (14.3%)	3,621 (14.3%)	1,586 (14.4%)	
Chromophobe	1,855 (5.10%)	1,261 (4.98%)	594 (5.38%)	
Not classified	9,024 (24.8%)	6,326 (25.0%)	2,698 (24.4%)	
Grade				0.537
I	2,772 (7.62%)	1,934 (7.64%)	838 (7.59%)	
II	12,432 (34.2%)	8,605 (34.0%)	3,827 (34.7%)	
III	7,630 (21.0%)	5,346 (21.1%)	2,284 (20.7%)	
IV	1,778 (4.89%)	1,219 (4.81%)	559 (5.06%)	
Unknown	11,753 (32.3%)	8,217 (32.5%)	3,536 (32.0%)	
Laterality				0.970
Left	18,015 (49.5%)	12,546 (49.5%)	5,469 (49.5%)	
Right	18,350 (50.5%)	12,775 (50.5%)	5,575 (50.5%)	
T				0.228
T1a	17,089 (47.0%)	11,907 (47.0%)	5,182 (46.9%)	
T1b	8,320 (22.9%)	5,844 (23.1%)	2,476 (22.4%)	
T2	3,475 (9.56%)	2,435 (9.62%)	1,040 (9.42%)	
T3	7,287 (20.0%)	4,998 (19.7%)	2,289 (20.7%)	
T4	194 (0.53%)	137 (0.54%)	57 (0.52%)	
N				0.561
N0	34,715 (95.5%)	24,161 (95.4%)	10,554 (95.6%)	
N1	1,650 (4.54%)	1,160 (4.58%)	490 (4.44%)	
Surgery				0.346
No	7,254 (19.9%)	5,092 (20.1%)	2,162 (19.6%)	
Local tumor excision	3,083 (8.48%)	2,132 (8.42%)	951 (8.61%)	
Partial nephrectomy	9,762 (26.8%)	6,835 (27.0%)	2,927 (26.5%)	
Radical nephrectomy	16,266 (44.7%)	11,262 (44.5%)	5,004 (45.3%)	
Chemotherapy				0.439
No/Unknown	34,096 (93.8%)	23,758 (93.8%)	10,338 (93.6%)	
Yes	2,269 (6.24%)	1,563 (6.17%)	706 (6.39%)	
Radiation				0.085
No/Unknown	35,260 (97.0%)	24,578 (97.1%)	10,682 (96.7%)	
Yes	1,105 (3.04%)	743 (2.93%)	362 (3.28%)	
Tumor size	49.7 (34.5)	49.6 (34.2)	50.0 (35.3)	0.426
Bone metastasis				0.063
No/Unknown	34,965 (96.2%)	24,378 (96.3%)	10,587 (95.9%)	
Yes	1,400 (3.85%)	943 (3.72%)	457 (4.14%)	
Brain metastasis				0.615
No/Unknown	36,025 (99.1%)	25,089 (99.1%)	10,936 (99.0%)	
Yes	340 (0.93%)	232 (0.92%)	108 (0.98%)	
Liver metastasis				0.666
No/Unknown	35,768 (98.4%)	24,900 (98.3%)	10,868 (98.4%)	
Yes	597 (1.64%)	421 (1.66%)	176 (1.59%)	
Lung metastasis				0.060
No/unknown	34,293 (94.3%)	23,917 (94.5%)	10,376 (94.0%)	
Yes	2,072 (5.70%)	1,404 (5.54%)	668 (6.05%)	

### Univariate and Multivariate Logistic Regression Analysis

In the training cohort, using the univariate logistic regression model to screen a risk factor for distant metastasis, the results showed that age, gender, race, year of diagnosis, marriage, tumor histologic type, histologic grade, T stage, N stage, tumor size, surgery, radiation therapy, chemotherapy is a risk factor for RCC distant metastases. The multivariate logistic regression model analyzed these variables. The results showed that race, tumor histological type, histological grade, T stage, N stage, tumor size, surgery, radiotherapy, and chemotherapy were independent risk factors for distant metastasis in elderly patients with RCC (Table 2). We can incorporate these risk factors into a nomogram to predict the risk of distant metastasis.

**Table 2:** Univariate and multivariate analyses of CSS in training set.

	**Univariate**			**Multivariate**		
	**HR**	**95% CI**	***P***	**HR**	**95% CI**	***P***
Age	1.02	1.01–1.02	<0.001	0.992	0.983–1	0.062
Race						
White	Reference					
Black	0.73	0.63–0.85	0	0.869	0.706–1.07	0.187
Other	1.14	0.97–1.35	0.11	1.358	1.072–1.72	0.011
Sex						
Male	Reference					
Female	0.87	0.8–0.95	<0.001			
Year of diagnosis						
2010–2014						
2015–2018	1.12	1.03–1.22	0.01			
Marital	Reference					
No						
Married	0.92	0.84–1	0.04			
Histologic type						
Clear cell	Reference					
Papillary	0.36	0.29–0.43	0	0.45	0.343–0.591	0
Chromophobe	0.21	0.14–0.32	0	0.262	0.154–0.445	0
Not classified	2.12	1.94–2.32	0	0.789	0.668–0.933	0.006
Grade						
I						
II	2.08	1.41–3.06	0	1.915	1.16–3.161	0.011
III	5.51	3.77–8.06	0	3.17	1.926–5.219	0
IV	20.69	14.02–30.55	0	5.565	3.315–9.342	0
Unknown	13.97	9.64–20.24	0	4.066	2.498–6.616	0
Laterality						
Left						
Right	0.95	0.87–1.03	0.22			
T						
T1a	Reference					
T1b	3.48	2.99–4.05	0	2.614	2.132–3.206	0
T2	11.14	9.55–12.98	0	4.639	3.616–5.953	0
T3	11.79	10.29–13.5	0	6.758	5.378–8.492	0
T4	74.58	51.57–107.85	0	14.282	8.417–24.236	0
N						
N0						
N1	21.81	19.18–24.81	0	3.858	3.219–4.622	0
Surgery						
No	Reference					
Local tumor excision	0.02	0.01–0.03	0	0.112	0.067–0.185	0
Partial nephrectomy	0.01	0.01–0.02	0	0.052	0.036–0.077	0
Radical nephrectomy	0.19	0.17–0.21	0	0.138	0.112–0.17	0
Chemotherapy:						
No/Unknown						
Yes	52.27	46.02–59.37	0	12.437	10.58–14.62	0
Radiation						
No/unknown						
Yes	83.54	67.18–103.87	0	28.853	22.196–37.506	0
Tumor size	1.03	1.02–1.03	0	1.006	1.004–1.008	0

### Construction and Validation of the Nomogram

**Figure 2:**
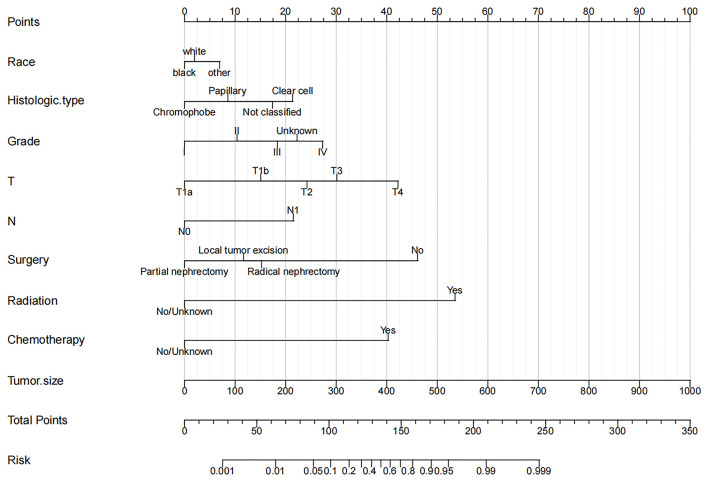
Nomogram for distant metastasis of elderly patients with RCC.

**Figure 3:**
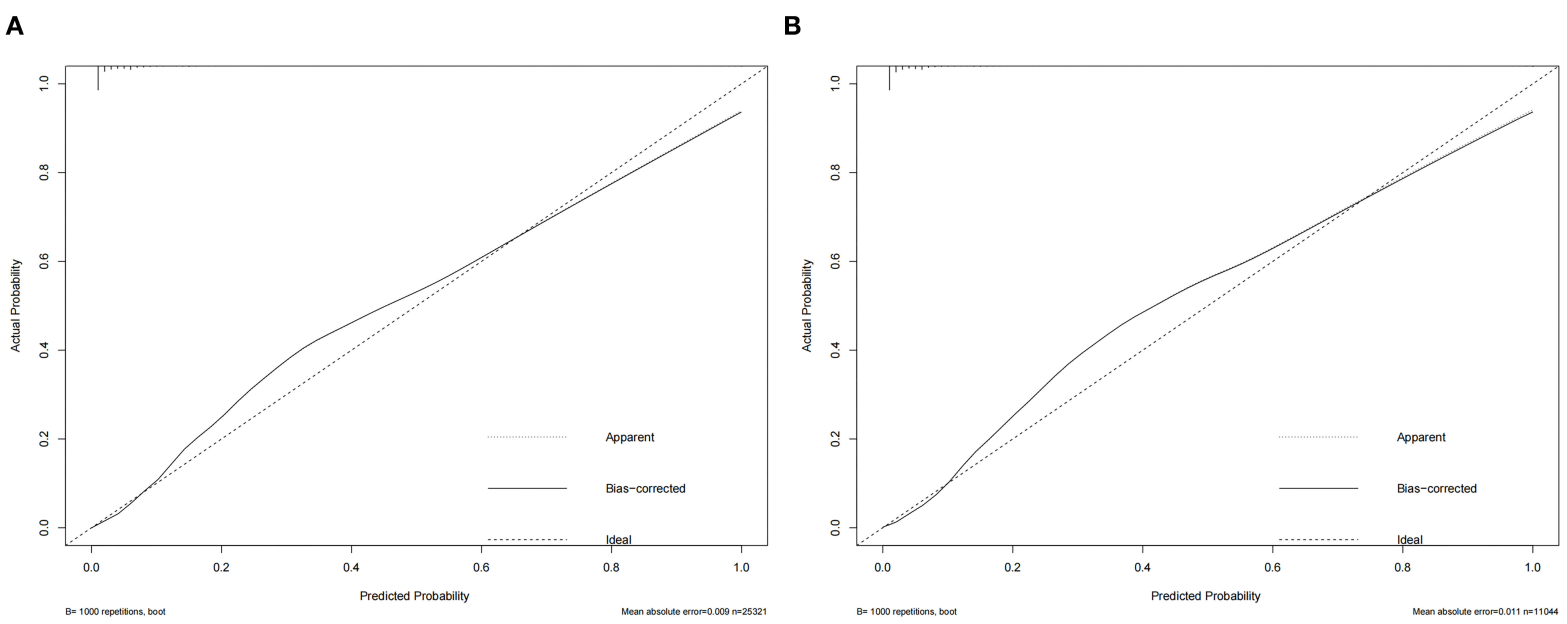
The nomogram's calibration curve in the training cohort **(A)** and the validation cohort **(B)**.

**Figure 4:**
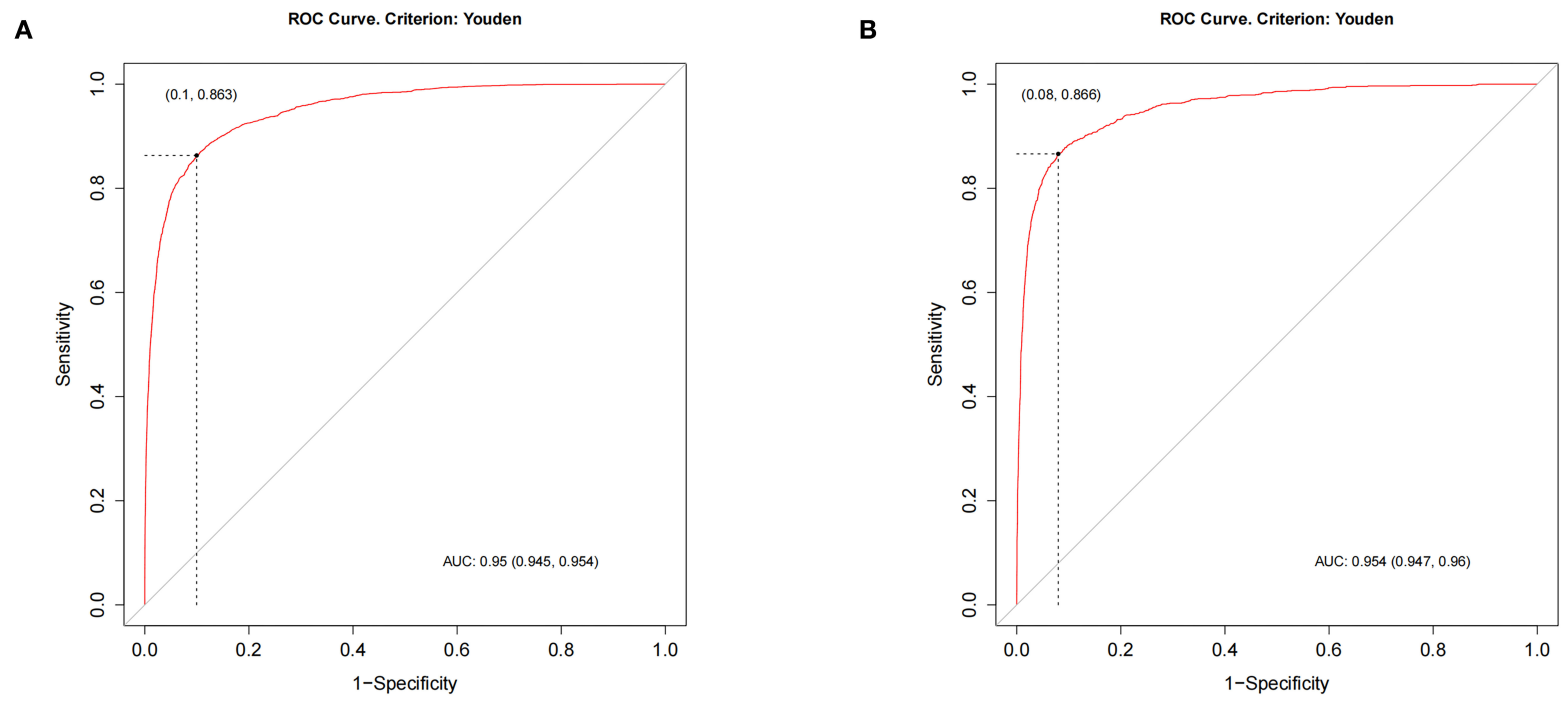
The ROC of the nomogram of training cohort **(A)** and validation cohort **(B)**.

Based on independent risk factors derived from univariate and multivariate screening, we constructed a new nomogram to predict the risk of distant metastasis in elderly patients with RCC (Figure 2). Tumor size was the most significant risk factor for distant metastasis, followed by radiotherapy, surgery, T stage, chemotherapy, histological grade, histological type, N stage, and race. Subsequently, calibration curves were used to validate the accuracy of the nomogram in the training cohort and the validation cohort, respectively. The results showed that the model's predicted values were consistent with the actual observed values (Figure 3). In the training and validation cohorts, the C-index was 0.949 (95% CI, 0.945–0.953) and 0.954 (95% CI, 0.947–0.960), respectively, indicating excellent accuracy. The AUC of the training cohort and the validation cohort also suggested excellent predictive power, with 0.95 (95%CI, 0.945–0.954) and 0.954 (95%CI, 0.947–0.96), respectively (Figure 4).

### Clinical Application of Nomogram

**Figure 5:**
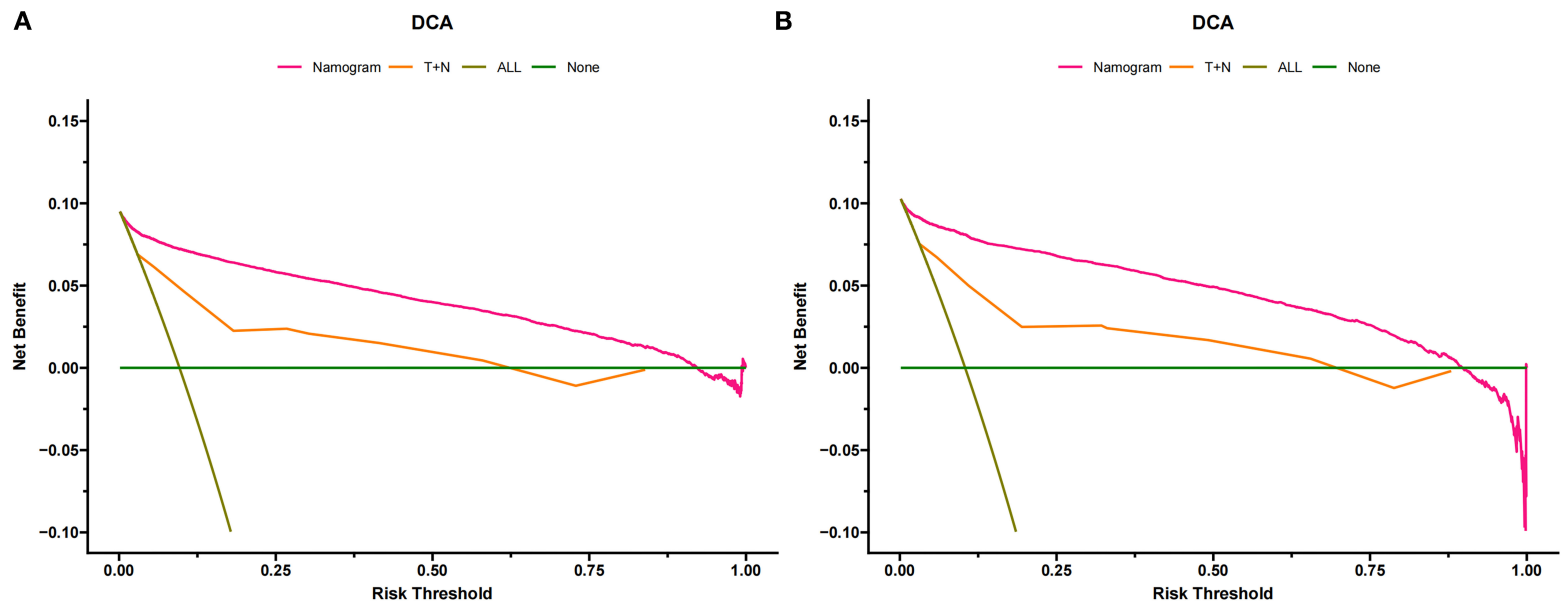
**(A)** Decision curves of the nomogram predicting distant metastasis in the training cohort and **(B)** the validation cohort. The x-axis is the risk threshold, and the y-axis is the net benefit. The purple line indicates no distant metastasis of the patient, and the blue line shows that all patients have metastasis. When the threshold probability is between 0 and 100%, the net benefit of the model is the largest.

**Figure 6:**
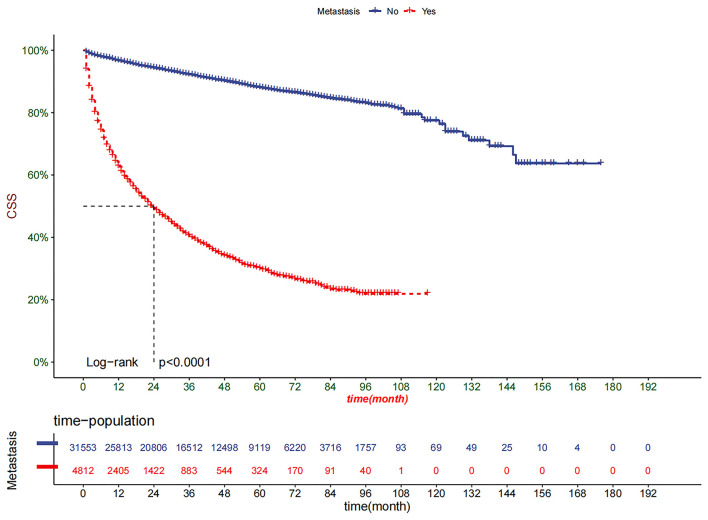
Kaplan–Meier curves of CSS for patients in the low-risk and high-risk groups.

**Figure 7:**
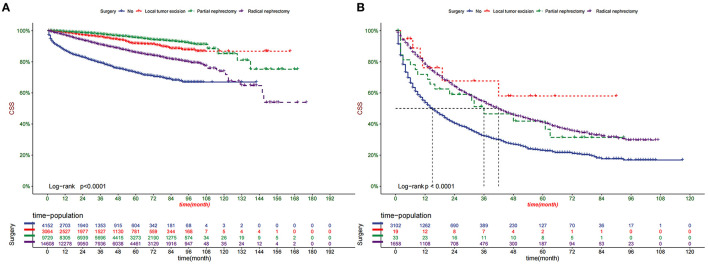
**(A)** Kaplan–Meier curves of CSS for patients with different surgery in the low-risk group and **(B)** the high-risk group.

DCA showed good clinical utility in training and validation cohorts (Figure 5). In addition, the nomogram has an obvious predictive advantage over TN staging. Patients in the training cohort and validation cohort were divided into the high-risk group (total score > 99.7) and low-risk group ( ≤ 99.7 overall). The K-M curve indicated that the 1-, 3-, and 5-year survival rates in the high-risk group were 0.627 (95% CI, 0.613–0.642), 0.406 (95% CI, 0.389–0.423), and 0.300 (95% CI, 0.282–0.320), respectively. The 1-, 3- and 5-year survival rates of patients in the low-risk group were 0.967 (95% CI, 0.964–0.969), 0.922 (95% CI, 0.919–0.926), and 0.880 (95% CI, 0.876–0.885), respectively (Figure 6). The results showed that patients in the high-risk group had a higher risk of distant metastasis and a worse prognosis. We analyzed surgical procedures in the high-risk and low-risk groups. In the low-risk group, patients with partial nephrectomy had the highest survival rate, followed by local tumor excision and radical nephrectomy. Patients without surgery had the worst survival rate (Figure 7A). In the high-risk group, where most patients did not undergo surgery or underwent radical nephrectomy, survival was significantly higher among patients undergoing surgery than among patients who did not undergo surgery (Figure 7B).

## Discussion

RCC is a high incidence tumor, with an estimated 330,000 patients worldwide every year (20), and RCC is still a severe problem that needs to be addressed in the United States (21). Non-metastatic RCC has a good prognosis after surgical treatment, including partial nephrectomy or radical nephrectomy (22). However, 30% of patients eventually develop metastatic RCC after surgical treatment, which is incurable (23). Currently, treatment for patients with metastatic RCC is minimal, including the use of cancer-targeted surgery and immune checkpoint inhibitor drugs to treat metastatic RCC. Still, the effect is inferior (24, 25). At present, CT is mainly used to detect the metastasis of RCC. Still, CT also has its inherent limitations, and its prediction of the risk of cancer metastasis is minimal (26). Therefore, it is necessary to accurately predict the risk of metastasis in patients, strengthening doctors' active monitoring of patients and preventing distant metastasis early.

At present, it is very limited in predicting distant metastasis of RCC. Hutterer et al. (27) collected renal cell carcinoma patients in 12 medical centers and established rosettes to predict distant lymph node metastasis, with an accuracy of 78.4%. Capitanio et al. (28) also established a prediction model to predict lymph node metastasis in RCC patients, with an accuracy of 86.9%. Marconi et al. (29) established a prediction model to predict the survival rate of patients with distant metastasis. Bai et al. (30) used MRI radiomic-based nomogram to predict distant metastasis of patients. Similarly, Zhao et al. (31) used CT radiomics to predict distant metastasis of patients. In addition, Li et al. (32) used patients in the SEER database to predict distant metastasis in RCC patients, but the C index was only 0.863. As far as we know, the current prediction model for distant metastases in RCC patients using nomograms has defects of a small number of patients and low prediction accuracy (Supplementary Table 1). Therefore, we aim to construct a highly accurate predictive model to predict distant metastases in elderly patients with RCC. The C-index of our prediction model is 0.949, which is much higher than the previous prediction models.

This study explored the risk factors for distant metastasis of elderly patients with RCC. We found that tumor size is the leading risk factor for distant metastasis of RCC, and the larger the tumor, the higher the risk of distant metastasis. Our results are similar to previous studies. Hutterer et al. (33) previously established a nomogram to predict distant metastasis of RCC and found that tumor size was a significant risk factor. Zastrow et al. (34) also found that tumor size was a risk factor for distant metastasis of renal cells. The risk of distant metastasis was significantly increased when tumor size was more significant than 3 cm. Li et al. constructed a nomogram to predict distant metastasis of RCC and found that tumors larger than 3 cm would increase the risk of metastasis (32).

Our study also found that tumor histological type and grade are important risk factors for distant metastasis. Patients with clear cell carcinoma of the kidney had the highest risk of distant metastasis, followed by papillary cell carcinoma of the kidney, and chromophobe cell carcinoma had the lowest risk of metastasis. Our results were similar to those of Zastrow et al. (34), who found that the risk of distant metastasis of renal clear cell carcinoma was the highest. In addition, we found that the degree of differentiation of RCC was associated with distant metastasis. The worse the differentiation, the higher the risk of metastasis. Previous studies have also found this phenomenon (34, 35). Because less differentiated tumors represent more aggressive biological behavior, which means they are more likely to metastasize far away.

In addition, the T stage and N stage are also crucial factors for tumor metastasis. Our study found that the higher the T stage, the greater the risk of tumor metastasis. This is understandable because the tumor invades the blood vessels and causes cancer cells to enter the bloodstream and metastasize. Lymph node metastasis is also a risk factor for distant metastasis of tumors. Previous studies have proved that regional lymph node involvement can lead to distant metastasis of tumors (6). They confirmed that regional lymph node involvement could increase the risk of distant metastasis by 50%.

In addition, surgery is also a key factor for distant metastasis of tumors. Patients without surgery are more likely to have distant metastasis than those with surgery because surgery can effectively remove or destroy cancer, thus reducing the chance of tumor metastasis. We divided patients into the high-risk metastatic and low-risk groups, and in the high-risk group, patients who underwent surgery had significantly improved survival outcomes. Patients with partial nephrectomy had the highest survival rate in the low-risk group. Because there are many complications in elderly patients, partial nephrectomy can remove the tumor with enough nephron remaining, resulting in a higher survival rate (36). Therefore, in the absence of contraindications, surgical treatment is still recommended to achieve a better outcome for patients at high risk of metastasis. For low-risk patients, partial nephrectomy is the best option.

This study explored the risk factors for distant metastasis in elderly patients with RCC. We used these risk factors to construct a graph to predict distant metastasis. After internal validation, the C-index could reach 0.95, and both the calibration curve and DCA showed the excellent accuracy of the prediction model. This prediction model can provide a practical, theoretical basis for patients' clinical decision-making and postoperative follow-up. To improve the survival rate and quality of life of elderly patients with RCC, we can better monitor the population at high risk of distant metastasis.

However, there are still some limitations to this study. First of all, this study is a retrospective study, so it is challenging to avoid selection bias. Therefore, prospective clinical trials are necessary to test the accuracy of the prediction model. Secondly, the SEER database lacks many related risk factors, such as comorbidity information, smoking, hypertension, BMI, etc. However, we included essential determinants such as tumor stage, histological type, and size so that our prediction model could achieve remarkably high accuracy. Finally, our prediction model is only validated internally, and further external validation is necessary to validate the accuracy and reliability of the model. Next, we plan to conduct a multi-center prospective clinical study to verify the accuracy of this prediction model.

## Conclusion

This study explored the risk factors for distant metastasis in elderly patients with RCC and found that race, tumor histological type, histological grade, T stage, N stage, tumor size, surgery, radiotherapy, and chemotherapy were independent risk factors for distant metastasis. We constructed a new nomogram to predict the risk of distant metastasis in elderly patients with RCC. With good accuracy and reliability, this nomogram can help doctors and patients to carry out active monitoring and follow-up of patients to prevent distant metastasis of tumors.

## Data Availability Statement

Publicly available datasets were analyzed in this study. This data can be found here: https://seer.Cancer.gov/.

## Ethics Statement

The data of this study is obtained from the SEER database. The patients' data is public and anonymous, so this study does not require ethical approval and informed consent.

## Author Contributions

JW and CZ designed the study. CZ, JW, JL, LJ, and XT collected and analyzed the data. JW drafted the initial manuscript. CZ, TM, JL, ZZ, and DH revised the article critically. CZ, JL, DH, LJ, and XT reviewed and edited the paper. All authors approved the final manuscript.

## Funding

This work was supported by Special Key Project of Chongqing Technology Innovation and Application Development (No. Cstc2019jscx-tjsbX0003).

## Conflict of Interest

The authors declare that the research was conducted in the absence of any commercial or financial relationships that could be construed as a potential conflict of interest.

## Publisher's Note

All claims expressed in this article are solely those of the authors and do not necessarily represent those of their affiliated organizations, or those of the publisher, the editors and the reviewers. Any product that may be evaluated in this article, or claim that may be made by its manufacturer, is not guaranteed or endorsed by the publisher.
