# Supplementary material for: Development and Validation of a Nomogram to Predict Distant Metastasis in Elderly Patients With Renal Cell Carcinoma
Source: Front Public Health. 2022 Jan 28;9:831940. doi: 10.3389/fpubh.2021.831940 (PMC8831843; doi:10.3389/fpubh.2021.831940)
Supplement: Supplementary file 1 [file Table_1.docx]

**Table S1. Literature of predictive models to predict distant metastasis in patients with renal cell carcinoma.**

| **Author** | **Year** | **prediction model** | **Number of patients** | **Data source** | **C-index** |
| --- | --- | --- | --- | --- | --- |
| Hutterer et al. [27] | 2007 | Nomogram for nodal metastases | 4658 | 12 centers | 0.784 |
| Capitanio et al. [28] | 2013 | Multivariable model for lymph node dissection | 1983 | A single tertiary care institution | 0.869 |
| Marconi et al. [29] | 2018 | predictive model of survival | 1108 | Multi-institutional European and North American data | 0.73 |
| Bai et al. [30] | 2020 | MRI radiomics-based nomogram for synchronous distant metastasis | 201 | A single center | 0.914 |
| Zhao et al. [31] | 2021 | CT radiomics for prediction of distant metastasis | 547 | Multi-institutional retrospective study | 0.861 |
| Li et al. [32] | 2021 | Prediction of distant metastasis | 37190 | SEER database | 0.863 |
